# Emerging psychosocial factors and work overload perceptions of Mexican university teachers and students working and studying from home during the COVID-19 pandemic

**DOI:** 10.3389/fpsyg.2024.1349458

**Published:** 2024-09-19

**Authors:** Belem Quezada Díaz, Imke Hindrichs, Doris Castellanos Simons

**Affiliations:** Centro de Investigación Transdisciplinar en Psicología (CITPsi), Universidad Autónoma del Estado de Morelos (UAEM), Cuernavaca, Mexico

**Keywords:** work overload, psychosocial factors, working from home, COVID-19, university teachers, university students

## Abstract

**Introduction:**

In Mexico, academic activities during the COVID-19 pandemic were conducted from home for over 2 years. Especially during the initial months of the pandemic, the lockdown conditions necessitated a reorganization and a new understanding of social dynamics. Therefore, this study aimed to explore the perceptions of university students and teachers regarding emerging psychosocial factors that either encouraged or hindered work and/or study from home during confinement, as well as their perceptions of work overload. Furthermore, the differences between students and teachers in the studied variables were analyzed.

**Method:**

A predominantly quantitative, cross-sectional, and correlational study was conducted with 108 participants (42.6% university teachers; 57.4% graduate or postgraduate students) who filled out an online questionnaire encompassing two open-ended inductors to identify the positive and negative aspects of working or studying from home and their frequency of perceptions, the COVID-19 Work Overload from Home Scale (ESTC-COVID-19), and questions about the hours per day devoted to different activities. The open responses were categorized by two independent groups of the research team; the emerging categories were then consensually agreed upon and further transformed into dummy and continuous variables. These variables and the results of the ESTC-COVID-19 were analyzed with SPSS 19 using Pearson's correlation coefficient, the Chi-squared test, and Student's *t*-test. The results identified 9 positive and 10 negative emerging psychosocial factors attributed to at least 10% of the sample's open answers. In addition, work overload correlated negatively with the emerging factor of “Making better use of time” and positively with “Work, school, and/or domestic activities overload;” moreover, students perceived more work overload than teachers.

**Discussion:**

Differences between students and teachers were observed in the following psychosocial factors: “Self-management,” “Comfort,” and “Enjoying home” (as positive factors) and “Domestic work” and “Interruptions, distractors, noise” (as negative factors), with students generally reporting more discomfort than teachers. The study analyzes these differences in relation to the demands and nature of the study and work activities undertaken by both groups, as well as the previous training of the skills and the resources required to carry them out.

## Introduction

In recent decades, the rapid evolution in the field of information and communication technologies has impacted work relationships and organizational structures. This shift, coupled with labor strategies implemented during the COVID-19 pandemic, led to work intensification and overload (Peiró and Soler, 2020; Avendaño, 2021). The advent of the Internet facilitated the continuation of the teaching and learning processes through online communication. However, this also increased the demand for constant connectivity and instant responses without pauses and delays. These new conditions for studying and working have introduced emerging psychosocial factors with both potential benefits and drawbacks for students and teachers in educational institutions, particularly within the university population.

The Committee on Occupational Health of the International Labor Organization and the World Health Organization (ILO/WHO) defines Psychosocial Factors at Work as those “interactions between and among work environment, job content, organizational conditions, and workers' capacities, needs, culture, personal extra-job considerations that may, through perceptions and experience, influence health, work performance, and job satisfaction” (International Labour Organization World Health Organization, 1986, p. 3). However, the conditions of those people who carried out their activities from home during the pandemic raise questions regarding the ILO/WHO concept since it separates the conditions of (and in) the organization from personal situations outside work, while, during confinement, everything was done in one place: personal and family activities, as well as working and studying, took place in the same dimension of space and time. Recognizing that a significant part of the academic work was already carried out from home even before the pandemic, Anwer (2020) and Sundari et al. (2020) pointed out that, with the lockdown, the boundaries between the spheres of life were completely blurred, particularly for female academics, leading possibly to perceived and objective work overload, especially for caregivers of scholars, who had to organize, support, and supervise children's homeschooling (Quezada et al., 2022a,b).

A conceptual framework that may be more consistent with the experience of working from home during the confinement is the proposal of Juárez and Camacho (2011), who define the psychosocial factors of work as “social facts occurring in the workplace that, in combination or dynamic interaction with an individual's traits and through bio-psychosocial pathogenic or salutogenic stress mechanisms, impact the health-disease process” (p. 202). These authors underline that the interrelationship among people, organizations, and work conditions results from a systemic, dynamic, and cyclic process of constant feedback. They also point out explicitly that not only negative factors but also salutogenic and protective factors must be considered, and both affect people and organizations. In their consideration not only of risk factors, the authors' proposal in their systemic framework resembles classical models, such as Karasek and Theorell's Demand-Control-Social Support model (Karasek and Theorell, 1990) and Siegrist's Effort-Reward Model (Siegrist, 1996) or the more recent and dynamic Job Demands-Resources theory of Bakker and Demerouti (2014), but it expands beyond their transactional and functional focus on specific demands and efforts, on the one hand, and resources and rewards, on the other hand, to take into account the peculiarities of the sociocultural and historical contexts, for example, of peripheral countries of the “global south” (i.e., outside of Europe or the USA), or non-white collar activities (as teaching and learning), inviting a more inductive approach that explores the point of view on the perceived reality of those who experience psychosocial factors, instead of applying predetermined and standardized models (Juárez et al., 2020). This framework, which represents the theoretical background of this article more accurately, adheres to the grounded theory tradition (Glaser and Strauss, 1967), which is centered on inductive categories that emerge from fieldwork rather than predetermined variables.

Gil-Monte (2012) states that psychosocial risk factors are those that originate from deterioration in task characteristics (e.g., high quantitative demands, monotony, and lack of control), organizational peculiarities (e.g., centralized leadership, poorly defined roles and functions), employment conditions (e.g., job insecurity, inadequate taxing conditions), and the organization of working time (e.g., long working days and flexible schedules). On the other hand, in addition to the perception of control, social support, and rewards (Karasek and Theorell, 1990; Siegrist, 1996), positive or salutogenic psychosocial factors include, for example, adequate material resources and tools, acknowledgment, and feedback (Juárez and Camacho, 2011), as well as the aspects and resources that promote personal growth, learning and development (Bakker and Demerouti, 2014), and positive relations with organizational leaders and family members, especially in times of crisis (Demerouti and Bakker, 2023).

Positive results and consequences of psychosocial factors can include overall wellbeing, satisfaction, personal development, motivation, and self-esteem. On the other hand, negative impacts can lead to work-related stress and occupational diseases, “especially the so-called psychosocial risks generated in the production processes, risks that negatively affect the relationships between the people with whom we work, the family, and the entire society” (Unda et al., 2016, p. 68). In this context, studying the emerging psychosocial factors in new or understudied work situations, such as working and studying from home in the specific distressing situation of the COVID-19 pandemic, seems relevant.

A significant number of studies have focused on the psychosocial risks of working from home even before the pandemic (Felstead and Henseke, 2017; Lott and Abendroth, 2023). This body of research grew enormously due to the nearly worldwide lockdown policies in 2020. However, fewer studies analyze both positive and negative psychosocial factors. Only a few studies across different occupations adopted an interactive perspective on the benefits and difficulties of working from home, and these studies were conducted before the COVID-19 lockdown policies (Oleniuch, 2021; Charalampous et al., 2022; Quezada et al., 2022b). Their findings outlined that working from home could increase job satisfaction, wellbeing, and organizational commitment, specifically if supervisors' trust and fairness were perceived and boundaries between personal life and work could be maintained (Lott and Abendroth, 2023). However, they also highlighted the risk of work intensification and difficulties in switching off (Felstead and Henseke, 2017). Other research focusing not only on emerging risks but also on protective factors and experiences of working from home during the pandemic restriction policies highlighted both positive and negative outcomes. Positive feelings included effectiveness (efficiency, work commitment, motivation, and concentration) and wellbeing (overall satisfaction, less exhaustion, and better work-life balance). However, negative feelings such as isolation, loneliness, boredom, sadness, anger, frustration, and stress were also reported, along with perceptions of expected availability, less comfort, detachment from work, and loss of sense and discipline (Oleniuch, 2021; Charalampous et al., 2022).

Furthermore, it is important to emphasize that Oleniuch (2021) found that the perceived benefits diminish with remote work experience while the sensation of difficulties increases. Moreover, Quezada et al. (2022a,b), in a study of the Mexican population, found that positive aspects include enhanced family interaction, especially with children, the comfort of staying home, no commuting, improved time management, and opportunities for self-management, adaptation, and new learning. However, they also identified several negative aspects, such as issues with schedules, workload, multitasking, interruptions, lack of necessary tools, connection problems, and challenges with online classes, as well as missing contact and interaction with others and work precarity. They also examined gender differences, finding that comfort and interruptions were more commonly associated with men, while interaction with children, adaptability, opportunities for new learning, and multitasking were more frequently associated with women.

Despite recognizing the differences between the activity of productive work intended as employment and the processes, roles, and functions of being students (Díaz-Barriga, 2021), it must be taken into account that university students and teachers are members of the same social institution where psychosocial factors are emerging and interacting. Furthermore, in psychosocial terms, working is considered as more than just (and sometimes even different from) employment and the teaching and learning process as more than just the transmission and acquisition of knowledge. Agreeing with Martín-Baró (1998), both studying and working are substantial activities that shape our identity as human beings: “Learning [...] is structuring a form of relation of a person with her/his environment, configuring a world where the individual occupies a place and materializes social interests. Working [...] is primarily and fundamentally making one oneself, transforming reality, finding or alienating oneself in one's task in the spider web of interpersonal and intergroup relations” (Martín-Baró, 1998, p. 168–169).

Regarding psychosocial aspects, although a very high number of studies were interested in the experiences of schools and universities, only a few studies could be identified that focus on the psychosocial factors and wellbeing of both university students and professors during the pandemic. An inquiry at two universities in the Middle East found that, during the confinement due to the COVID-19 outbreak, good family relationships, physical comfort, and goal achievement had positive effects on academic wellbeing, while self-reported depression, headaches, enhanced eating, and sleeping affected the participants negatively (Al-Sabbah et al., 2021). Members (teachers, researchers, staff, residents, and interns) of a veterinarian university in Canada reported more quantitative demands, burnout, stress, and depressive symptoms and less recognition and sense of community than the Canadian norm, as well as deterioration during the 1st months of the pandemic, with restrictions of emotional demands, health and wellbeing, and work-life conflict (McKee et al., 2021). While these two studies did not indicate differences between students and university professors, an inquiry at the University of Barcelona found that students were more affected by temporary employment, negative interaction between work and home and vice versa (while rating lower in positive home and work interaction), and teleworking itself, and they reported more interpersonal conflicts and negative affective states than the rest of the university population (Romeo et al., 2021).

At this point, it is important to note that, in Mexico, teleworking was not recognized in labor laws and regulations in 2020. It was not until mid-2023 that the official Mexican standard “Teleworking Conditions of Safety and Health at Work” (Ley Federal del Trabajo, 2023) was established. This standard acknowledges teleworking as a form of subordinate work organization that allows for paid activities to be performed outside the traditional workplace, which does not require the physical presence of the worker since information and communication technologies are used for contact and control between the worker and the employer. While this legal recognition provides a framework for implementing telework properly, bad practices persist among leaders and employers (Online Career Center, 2023), and many companies either disregard or remain unaware of the telework regulations (Salas, 2024). According to January 2024 statistics, 13 million people are working remotely or teleworking in Mexico (Becerra, 2024), including those starting during the COVID-19 pandemic, as well as those working under flexible or hybrid arrangements. This form of work is also used temporarily during emergencies such as health crises or shortages of public resources, among others. Given this context, it is relevant to understand the psychosocial processes involved in teleworking to enhance the adaptation and improvement of government regulations and, above all, to ensure that workplaces implement appropriate measures in their practice.

Regarding the educational conditions during the confinement policies due to the COVID-19 pandemic, UNESCO (2020) reported that, as of 30th March 2020, 166 countries worldwide had closed schools and universities. The Economic Commission for Latin America (ECLA), known by its Spanish acronym CEPAL, informed that, by August 2020, 29 Latin American countries had implemented strategies to ensure the continuity of schooling from a distance. These strategies include both synchronous and asynchronous activities, as well as offline and online methods. Additionally, they utilized Internet platforms and more traditional media, such as broadcasting classes on national television (Comisión Económica para América Latina y el Caribe, 2020).

Particularly in Mexico, since March 2020, university classes have been conducted mostly online for about 2 years. The Federal Government implemented different actions to reduce contagion, including national confinement (though not as controlled as in other regions), the temporary suspension of non-essential activities, and a significant shift to remote work from home. In April, the Secretary of Public Education launched the “Learn from Home” program, indicating that formal schooling at all educational levels had to be done from home (SEP, 2020).

In any case and at all educational levels, online classes represented a challenge involving the transfer of traditional activities to a virtual environment. Often, both teachers and students lack the necessary skills and, in some cases, the means to use online platforms, making it difficult, and sometimes impossible, to adapt pedagogical contents to meet learning objectives. As Hurtado (2020) pointed out, these challenges extended to the abrupt shift to distance learning; the struggle to adapt to new technologies, which required additional learning for both students and educators; issues with connectivity, access, and infrastructure; impacts on mental health; adjustments to the virtual environment, including motivation, participation, and interaction with peers; interruptions and loss of social and extracurricular activities; and economic concerns, among others.

Regarding universities, Alcántara (2020) noted that higher education institutions worldwide mandated an abrupt transition to virtual education, even when there was insufficient infrastructure and preparation among teachers and students. This sudden shift highlighted the digital divide and socioeconomic issues affecting both groups. Universities faced the challenge of rapidly organizing teacher training programs, often encountering significant difficulties. Additionally, they had to implement urgent measures for economic and socioemotional support for students, among other critical needs.

In the particular case of Mexico, where this research was conducted, and Latin America in general, no specific studies that address university teachers and students regarding this study's goal were found. However, there is separate research on teachers and students in the region. Studies with teachers in Latin American countries since the beginning of the pandemic have reported several psychosocial risk factors in the workplace: adaptation to ICTs, increased work at home, fear of contagion (Robinet-Serrano and Pérez-Azahuanche, 2020), home and its environment characteristics, influence of the extra-work environment, control over work, poor rewards (Robalino, 2023), high job demands (Godínez-Tovar et al., 2023; Robalino, 2023), problems with household members (Avila-Valdiviezo et al., 2021), mental overload, work competence, and inadequate resolution of planned problems (Godínez-Tovar et al., 2023). Although psychosocial risk factors are more extensively documented with scientific evidence, there are also reports of positive psychosocial factors and wellbeing. For example, Soto-Crofford and Deroncele-Acosta (2021) report collaborative work, assertive communication, self-care habits, and spirituality.

On the other hand, in Mexico, Villagrán et al. (2022) investigated the perceptions of teachers from a public university and a teacher training college in Jalisco regarding the shift from face-to-face to virtual mode at work and the psychosocial risks arising from the COVID-19 pandemic. The study identified several factors significantly associated with teachers' negative evaluations, including the technostress caused by the demands of the sudden transition from face-to-face to virtuality and the incorporation of ICTs, the pressure to use these technologies, and the need to diversify the education strategies, which is associated with the perception of not being prepared or trained.

In the context of remote or online classes, university students have also been significantly affected. Previous reports indicate increases in stress levels (Martinez Arriaga et al., 2021; Robles et al., 2021; Romero et al., 2022), as well as heightened anxiety and sadness (Martinez Arriaga et al., 2021), uncertainty, fear, and even academic dropout (Romero et al., 2022). Robles et al. (2021) highlighted family conflicts, such as family violence, grief from losing family members, conflicts due to the invasion of privacy, and economic difficulties impacting students. Additionally, Rocha-Ibarra et al. (2023) found a correlation between the number of hours spent on academic activities at home using a computer and eye problems among Mexican university students from the University of Guanajuato.

Similarly, Gazca-Herrera and Mejía-Gracia (2022) study involving students from the University of Veracruz highlighted that the shift to virtual learning poses challenges for teachers and students not only due to inadequate preparation for online teaching and learning activities but also because of limited access to technology and its use at home. According to Martinek et al. (2021), these factors were predictors of student frustration in distance education and online learning. On the other hand, Martinek et al. (2021) emphasized that rapid and unexpected transition to virtual learning led to changes in the perception of teaching and learning processes, workload and time management, social relationships, and students' self-assessment of their competencies and ability to continue with previously projected trajectories. Furthermore, Tavera-Fenollosa and Martínez Carmona (2021) conducted a qualitative study with UNAM students, revealing significant negative emotional impacts from the interruption of face-to-face interactions on relationships and interactions with other key individuals in students' lives.

The objective of this study was to explore university students' and teachers' perceptions of emerging psychosocial factors that encourage, motivate, hinder, or stress work or study from home during confinement, as well as their perception of work overload, further analyzing the differences between students and teachers in their prevalences of experiencing emerging psychosocial factors and their perceptions of work overload. The findings from this research can enhance the understanding of difficulties affecting academic performance and the teaching and learning process, help identify psychosocial risk factors to prevent mental health problems, and inform measures to improve the effectiveness of this emerging work model. This is particularly relevant as some emerging psychosocial factors may continue to impact the university population even after the return to on-site schooling, and recognizing that, with or without the pandemic, a significant amount of academic activity for professors and students will be conducted at home is crucial.

## Materials and methods

The study employed a predominantly quantitative approach with a qualitative component, utilizing a mixed analysis technique. Open-ended questions were analyzed using qualitative principles but were also quantified based on the participants' frequency of experience to estimate their perceived relevance (Juárez et al., 2020), as described in more detail in the following paragraphs. Participants were selected through convenience and snowball sampling. The criteria for participation included being over 18 years old, residing in Mexico, and working and/or studying from home due to COVID-19 pandemic confinement period. This study focused on a sub-sample (*N* = 108) of academics, comprising university students (*N* = 62) and teachers (*N* = 62), representing 33.64% of the entire sample.

The protocol of the entire research project was approved by the Research Ethics Commission of the Transdisciplinary Research Center in Psychology of the Autonomous University of Morelos, and participation was voluntary and anonymous. Data were collected from 25th May 2020 to 6th August 2020 by an online survey. Invitations were sent by the research team, as well as colleagues' and friends' virtual social networks and e-mail lists. The sample was non-probabilistic by convenience.

### Instruments

In order to explore the psychosocial factors from the participants' perceptions and experiences during the 1st weeks and months of confinement due to the pandemic outbreak in Mexico, the proposal of the mixed data analysis technique of Juárez et al. (2020) was adapted, formulating the following two open-ended inductors: *1. Indicate three to five aspects that you like the least, which affect the execution of your work, or which cause you tension or discomfort in your current work situation* and *2. Mention three to five aspects that you like the most, which motivate you, or which cause you enthusiasm in the execution of your work in your current work situation*. The participants were additionally asked to indicate the frequency of these aspects during their confinement at home on a Likert scale from 1 (*rarely or never*) to 5 (*always, every day*).

Furthermore, the *COVID-19 Work Overload from Home Scale* (ESTC-COVID-19 by its acronym in Spanish), developed and validated for the Mexican population by Quezada et al. (2022a,b), was applied. It intended to evaluate the qualitative work overload (considering spillover between productive and domestic activities) and the lack of balance between the demands and the control to manage them, i.e., the participants' subjective perceptions based on their evaluation of their capabilities, skills, and competencies to fulfill their tasks. The scale consisted of seven items (e.g., *I feel overwhelmed by work at home and domestic tasks*) and was responded with a Likert scale from 1 (*never*) to 5 (*always*). The analyses revealed a Cronbach's alpha of 0.778.

Finally, as a complementary and more objective, although self-reported, indicator of overload, participants were asked how many hours a day they were devoted to working and/or studying from home, domestic duties, caregiving, and helping with children's homework. Some sociodemographic indicators (sex, age, marital status, parenthood, and occupation) were also collected so that the sample could be characterized.

### Data analysis

The answers to the open inductors that aimed to explore emerging positive and negative psychosocial factors were analyzed following the suggestions from Juárez et al. (2020). Corresponding to the grounded approach (Glaser and Strauss, 1967) of this proposal, the answers were coded inductively (Corbin and Strauss, 2014) into macro- and micro-categories. To provide constant comparison and to avoid biases in the codification, this process was first conducted by two independent teams, who then came together (virtually in video calls and using online spreadsheets due to the confinement) to find consensus on the categorization and define the emerging categories. Additionally, one team was formed by experts in psychosocial factors, while the other team consisted of interns (students with bachelor's degree in psychology), so professional expertise and biases could be balanced with a fresher look at the participants' answers.

Once the categories were defined, owing to the indications of frequency of exposure to the aspects mentioned by participants, they could be transformed not only into dummy variables (i.e., whether a participant mentions or not an aspect categorized as a certain factor) but also into continuous variables (i.e., considering the indicated frequency of exposure to a certain aspect). Finally, these resulting variables of an inductive nature were matched with the database of the remaining deductive variables addressed by the study.

The resulting database was analyzed using SPSS 19 software, enabling descriptive statistics and exploratory correlations among the variables to be carried out and the differences between students' and teachers' perceptions of work overload to be analyzed, as well as their experiences with and exposure to emerging psychosocial factors: differences between means were analyzed using Student's *t*-test, and the association between the ESTC-COVID-19 scale and the emerging psychosocial factors was explored using Pearson's correlation coefficient. Furthermore, the chi-squared test was applied in order to explore if some of the emerging psychosocial factors, treated as a dummy variable (a participant did = 1 or did not = 0 give answers labeled with a category, not considering the frequency of its perceptions), were associated specifically with the nominal variable student or teacher.

## Results

### Sample characteristics

Out of the 108 participants, 42.6% (*N* = 46) were university teachers, and 57.4% (*N* = 62) were graduate or postgraduate students. They were distributed across 19 of the 32 federal states of Mexico. Table 1 provides detailed sociodemographic information, including age, marital status, and parenthood, categorized by participant type and sex.

**Table 1:** Sociodemographic information.

	***N***	**%**	**Sex**	***n***	**Mean age**	**Age range**	**Marital status**	
							**With a partner**	**Single or separated**
Students	62	57.4%	Female	46	28	18–47 years	15	30
			Male	16	26	18–35 years	2	14
Teachers	46	42.6%	Female	37	45	27–67 years	17	20
			Male	9	41	27–64 years	5	4

Additionally, 42 students (32 women, 10 men) stated that they were only studying, while 20 students mentioned they were both studying and working (14 women, six men). Furthermore, 33 students (25 women, eight men) had some kind of scholarship, while the remaining 27 students (19 women, eight men) did not.

### Categorization of emerging psychosocial factors

In the overall sample, 426 answers to the prompt to list positive aspects and 481 answers to the prompt to list negative ones were given. Following the above-detailed procedures (Juárez et al., 2020), the positive aspects were categorized into 44 emerging macro categories and the negative ones into 50. For the aim of the present study, the descriptive results of the positive and negative emerging categories and variables, which included the answers of at least 10% of the 108 academic participants, are presented as follows.

### Positive psychosocial factors

As detailed in Table 2, nine positive psychosocial factors that group the answers of at least 10% of participants could be identified: *Family interaction, Self-management, No need for commuting, Nutritional wellness, Adaption and new learning, Comfort, Enjoying home, Teleworking*, and *Making better use of time*. The mean frequency of experiencing the factors oscillated between 3.5 and 4.6 on the 1–5 Likert scale.

**Table 2:** Positive psychosocial factors by number of participants and frequency of experience.

**Macro-categories**	***N***	***%***	**Mean of experience**	**SD**
Family interaction	28	25.93	4.5	0.73
Self-management	20	18.52	3.9	1.03
No need for commuting	19	17.59	4.4	0.83
Nutritional wellness	17	15.74	4.6	0.82
Adaption and new learning	16	14.81	4.2	0.65
Comfort	16	14.81	4.4	0.51
Enjoying home	13	12.04	4.3	0.72
Teleworking	12	11.11	4.6	0.79
Making better use of time	11	10.19	3.5	1.03

N, number of participants that gave at least one answer labeled by a category.

The category *Family interaction* (*N* = 28) includes answers referring to the possibility of communicating, living together, enjoying, having more time, solving problems, and giving attention to one's family. Some textual quotes from the participants' answers were: “Time with my parents because I [normally] live far from them,” “I can be in touch with my parents,” and “Visiting my family.” The category *Self-Management* (*N* = 20) refers to the sensation or experience of having more control and the possibility to manage different aspects of life (working, household, personal issues, etc.), as well as the time to organize one's schedule in an independent and self-taught way, for example: “Better organization,” “Possibility to do other activities at home,” and “I can work with my own schedule.” *No need for commuting* (*N* = 19) is comprised of answers that refer to the benefits related to not needing to travel from home, avoiding wasting time, traffic, or public transportation (for example: “I do not deal with hours of commuting;” “Less transportation time;” and “Not to have to waste time in the commute to the university”). *Nutritional wellness* (*N* = 17) reassembles the benefits of eating at home, associated with the possibility of eating together, not going to another place to eat, eating at one's own schedule, and eating more varied, better-cooked, and healthier food. It is the positive factor with the highest mean score to be experienced. *Adaption and new learning* (*N* = 16) refers to the opportunities for new experiences, the possibility of work reorganization in the new reality, and new learning achieved during the confinement, including knowledge improvement, new training options, new ways of working, the acquisition of digital skills, and access to online learning. The category of *Comfort* (*N* = 16) includes answers that refer to the opportunity to work with comfortable clothing, not getting dressed up, or having the chance to take breaks. The category *Enjoying home* (*N* = 13) includes the pleasure of being at one's own home, taking care of the house and improving it, spending more time in it, and enjoying the place. The answers categorized as *Teleworking* (*N* = 12), the factor with the highest mean frequency of being experienced, refer to the benefit of working from home for the space, the available tools, and the convenience of working online, being more productive, and avoiding contagion. Finally, the category *Making better use of time* (*N* = 11) has the lowest mean frequency of being experienced. It bundles the perceptions of better time management and having more time for recreation, self-care, working, and studying.

### Negative psychosocial factors

Table 3 shows the 10 negative psychosocial factors that emerged in at least 10% of the participants' answers: *Work overload, school and/or domestic activities; Schedules; Online classes; Interruptions, distractors, noise; Problems with Internet services; Confinement; Lack of physical and/or affective contact/interaction; Domestic work; Family environment*; and *Stress*. The mean frequency of experiencing the emerging factors shifts from 3.2 to 4.5, which is a slightly lower range than the one observed for positive factors.

**Table 3:** Negative psychosocial factors by number of participants and frequency of experience.

**Macro-categories**	***N***	***%***	**Mean of experience**	**SD**
Work, school, and/or domestic activities overload	29	26.85	4.3	0.87
Schedules	25	23.15	4.4	0.58
Online classes	17	15.74	4.2	0.58
Interruptions, distractors, noise	17	15.74	4.2	0.66
Problems with Internet services	17	15.74	3.2	1.01
Confinement	12	11.11	4.1	0.99
Lack of physical and/or affective contact/interaction	12	11.11	4.1	0.79
Domestic work	11	10.19	4.3	1.00
Family environment	11	10.19	3.6	0.72
Stress	11	10.19	4.5	0.68

N, number of participants that gave at least one answer labeled by a category.

The category that groups answers from most participants was *Work, school, and/or domestic activities overload* (*N* = 29), referring to experiences of increased load in paid or productive work, school activities, and/or house chores. Among the textual answers were, for example, “Increase of workload” and “Homework overload;” one answer also explains the reason for the discomfort: “Workload. Due to training and instructions from the management staff, I have more work to do since I don't only have to review classes and grades but also develop work that was previously conducted by the administrative staff.” Answers labeled with the category *Schedules* (*N* = 25) indicated problems in managing new schedules and time management during teleworking for individuals and organizations, e.g., extension of working hours, lack of clarity in schedules, feelings of having to stay available for work all the time, time being not enough for neither work nor personal activities, as well as not achieving good time management or organization. Some specific answers follow: “Not having a schedule,” “More working hours,” and “There is no fixed schedule to communicate, so they can ask for school assignments at any time of the day.” The category *Online Classes* (*N* = 17) concentrates on answers about the discomfort of schooling at a distance perceived by both students and teachers, e.g., “Little concern from teachers,” “Homework without explanation,” and “Problems communicating with students of limited economic resources.” Participants whose answers were categorized as *Interruptions, distractors, noise* (*N* = 17) mentioned agents that interfere with the performing of work or school activities at home, as interruptions and distractions by other people at home, as well as noise inside and outside the house and in the neighborhood: “There is a lot of noise at home;” “Noise from neighbors;” “Noise from household members while taking online classes.” The category *Problems with Internet services* (*N* = 17) encompasses inconveniences resulting from problems with the Internet service, such as connection failures or slow service. For instance, although 15.74% of the participants gave answers labeled with this category, it has the lowest average frequency of being experienced. The category *Confinement* (*N* = 12) concentrates on answers that directly express the discomfort of being confined, such as isolation, enclosure, not going out, and/or feeling lonely, while answers categorized as *Lack of physical and/or affective contact/interaction* (*N* = 12) indicate explicitly the trouble of not having physical or affective contact and interaction with others (family, friends, students, teachers, or colleagues). The category *Domestic work* (*N* = 11) refers to the amount of house chores, such as cleaning, laundry, dishwashing, as well as the responsibility of organizing these tasks. Answers labeled as *family environment* (*N* = 11) refer to the difficult family dynamics during the confinement, patience in conflicts, stressful interaction, and a lack of cooperation between family members. Finally, the category *Stress* (*N* = 11), which has the highest observed mean score in its experimentation, groups answers that refer explicitly to the perceived tension and stress during the confinement.

### Work overload from working or studying from home during the confinement due to COVID-19 and its relation with emerging psychosocial factors

Table 4 shows the means and standard deviations of the ESTC-COVID-19, which measured perceived work overload during the Mexican lockdown, and their differentiations between students and teachers. The differences between means are statistically significant (*p* = 0.023), indicating that students tend to perceive overload on average more frequently than teachers. Nevertheless, the more objective data of study from home showed that 22 teachers (47.83%) studied more than 8 h a day and 19 students (30.65%) studied only either 2–4 or 4–6 h; for house chores, both groups indicated spending 2–4 h daily, accounting for 43.48% of teachers and only 30.64% of students.

**Table 4:** Mean and standard deviation SD of the ESTC-COVID-19 scale.

**Participants**	***N***	**%**	**Mean**	**SD**
Students	62	57.40	3.58	0.60
Teachers	46	42.60	3.31	0.60
Overall	108	100%	3.46	0.61

Furthermore, as shown in Table 5, only two factors correlated positively with the ESTC-COVID-19 at a statistically significant level: *Making better use of time* negatively and *Work, school, and/or domestic activities overload*. On the one hand, the more frequently the 11 participants (six students, five teachers) who gave answers categorized as *Making better use of time* indicated to experiment with this aspect, the less qualitative work overload they perceived. On the other hand, the 29 participants (15 students, 14 teachers) who indicated answers categorized as *Work, school, and/or domestic activities overload* experimented with this aspect more frequently, as they also perceived more quality work overload, indicating an interesting semantical concordance between the theoretical conception of the ESTC-COVID-19 and the inductive analysis.

**Table 5:** Pearson correlation between the ESTC-COVID-19 scale and emerging psychosocial factors.

		**Making better use of time**	**Work, school, and/or domestic activities overload**
ESTC—COVID-19	Pearson correlation	−0.694^*^	0.552^**^
	Sig. (bilateral)	0.018	0.002
	*N*	11	29

^*^Correlation is significant at the 0.05 level (two-tailed).

^**^Correlation is significant at the 0.01 level (two-tailed).

Only significant associations are reported.

### Differences between students and teachers in the perceptions of psychosocial factors

In accordance with the abovementioned differences between students and teachers in their work overload perceptions, if there were differences in how they perceived the emerging psychosocial factors was also explored.

The possibility of treating the emerging factors as continuous variables not only permitted the correlation mentioned above with the ESTC-COVID-19 scale but also allowed the implementation of Student's *t*-test to see differences in the mean frequency with which students and teachers experienced certain factors.

Table 6 reports the differences in the frequency of perceptions of positive psychological factors. It can be seen that, on average, teachers enjoy *Self-management* and the *Comfort* of working from home more often than students do (although equal variances could not be assumed in both cases).

**Table 6:** *T*-test of differences between teachers' and students' perceptions of positive emerging psychosocial factors.

**Emerging positive factors**		***N***	**Mean**	**SD**	***t***	***P***
Family interaction	Students	17	4.32	0.85	−1.10	0.28
	Teachers	11	4.64	0.50		
**Self-management**	**Students**	**14**	**3.46**	**0.97**	**−4.44** ^*^	**0.00**
	**Teachers**	**6**	**4.83**	**0.41**		
No need for commuting	Students	12	4.58	0.51	0.91^*^	0.39
	Teachers	7	4.14	1.21		
Nutritional wellness	Students	9	4.78	0.44	1.12^*^	0.29
	Teachers	8	4.31	1.10		
Adaption and new learning	Students	8	4.31	0.70	0.56	0.59
	Teachers	8	4.13	0.64		
**Comfort**	**Students**	**12**	**4.25**	**0.45**	**−5.74** ^*^	**0.00**
	**Teachers**	**4**	**5.00**	**0.00**		
Enjoying home	Students	4	4.00	0.82	−0.88	0.40
	Teachers	9	4.39	0.70		
Teleworking	Students	5	4.40	0.89	−0.66	0.52
	Teachers	7	4.39	0.76		
Making better use of time	Students	6	3.50	1.05	0.15	0.88
	Teachers	5	3.40	1.14		

Significant statistical differences are in bold.

^*^Equal variances could not be assumed.

The differences in the frequency of perceiving negative factors are indicated in Table 7. The only difference found pertained to *Domestic work*: the four teachers who provided responses categorized under this label reported experiencing it consistently (5 = “always, every day”), and statistically more significantly than the seven students whose responses fell under the same category, with an assumption of unequal variances.

**Table 7:** *T*-test of differences between teachers' and students' perceptions of negative emerging psychosocial factors.

**Emerging negative factors**		***N***	**Mean**	**SD**	***t***	***P***
Work, school, and/or domestic activities overload	Students	15	4.20	0.86	−0.48	0.64
	Teachers	14	4.36	0.91		
Schedules	Students	13	4.37	0.55	−0.36	0.72
	Teachers	12	4.46	0.66		
Online classes	Students	7	4.21	0.39	−0.12	0.91
	Teachers	10	4.25	0.72		
Interruptions, distractors, noise	Students	14	4.29	0.61	0.66	0.52
	Teachers	3	4.00	1.00		
Problems with Internet services	Students	12	3.00	1.04	−1.12	0.28
	Teachers	5	3.60	0.89		
Confinement	Students	9	4.11	0.78	0.11^*^	0.92
	Teachers	3	4.00	1.73		
Lack of physical and/or affective contact/interaction	Students	8	3.88	0.83	−1.33	0.21
	Teachers	4	4.50	0.58		
**Domestic work**	**Students**	**7**	**3.86**	**1.07**	**−2.83** ^*^	**0.03**
	**Teachers**	**4**	**5.00**	**0.00**		
Family environment	Students	7	3.86	1.22	0.79	0.45
	Teachers	4	3.25	1.26		
Stress	Students	6	4.15	0.75	0.12	0.32
	Teachers	5	4.89	0.44		

Significant statistical differences are in bold.

^*^Equal variances could not be assumed.

Finally, as shown in Table 8, exploring the association between psychosocial factors and being a student or teacher, the only prevalence between students and teachers for positive psychosocial factors was found in the category *Enjoying home* (*p* = 0.039; correct residual > 1.96): This variable appears to be statistically significantly related to teachers.

**Table 8:** Chi-square test between numbers of students and teachers who gave answers codified as a positive psychosocial factor.

**Emerging positive factor**		**Observed count**	**Expected count**	**Corrected residual**	**Sig**.
Family interaction	Student	17	16.1	0.4	0.825
	Teacher	11	11.9	−0.4	
Self-management	Student	14	11.5	1.3	0.316
	Teacher	6	8.5	−1.3	
No need for commuting	Student	12	10.9	0.6	0.619
	Teacher	7	8.1	−0.6	
Nutritional wellness	Student	9	9.8	−0.4	0.791
	Teacher	8	7.2	0.4	
Adaption and new learning	Student	8	9.2	−0.6	0.589
	Teacher	8	6.8	0.6	
Comfort	Student	12	9.2	1.5	0.172
	Teacher	4	6.8	−1.5	
**Enjoying home**	**Student**	**4**	**7.5**	**−2.1**	**0.039**
	**Teacher**	**9**	**5.5**	**2.1**	
Teleworking	Student	5	6.9	−1.2	0.354
	Teacher	7	5.1	1.2	
Making better use of time	Student	6	6.3	−0.2	0.542
	Teacher	5	4.7	0.2	

Significant statistical differences are in bold.

On the other hand, as reported in Table 9 for the negative psychosocial factors, statistical significance was found within the following categories: *Interruptions, distractors, noise* (*p* = 0.032) and *Confinement* (*p* = 0.023). Expressing discomfort from *Interruptions, distractors, noise* appears to be associated with being a student.

**Table 9:** Chi-square test between numbers of students and teachers who gave answers codified as a negative psychosocial factor.

**Emerging negative factor**	**Sex**	**Observed count**	**Expected count**	**Corrected residual**	**Sig**.
Work, school, and/or domestic activities overload	Student	15	16.6	−0.7	0.515
	Teacher	14	12.4	0.7	
Schedules	Student	13	14.4	−0.6	0.646
	Teacher	12	10.6	0.6	
Online classes	Student	7	9.8	−1.5	0.183
	Teacher	10	7.2	1.5	
**Interruptions, distractors, and noise**	**Student**	**14**	**9.8**	**2.3**	**0.032**
	**Teacher**	**3**	**7.2**	**−2.3**	
Problems with Internet services	Student	12	9.8	1.2	0.291
	Teacher	5	7.2	−1.2	
**Confinement**	**Student**	**9**	**6.9**	**1.3**	**0.023**
	**Teacher**	**3**	**5.1**	**−1.3**	
Lack of physical and/or affective contact/interaction	Student	8	6.9	0.7	0.552
	Teacher	4	5.1	−0.7	
Domestic work	Student	7	6.3	0.4	0.756
	Teacher	4	4.7	−0.4	
Family environment	Student	7	6.3	0.4	0.756
	Teacher	4	4.7	−0.4	
Stress	Student	6	6.3	−0.2	0.542
	Teacher	5	4.7	0.2	

Significant statistical differences are in bold.

## Discussion

This research explored the emerging positive and negative psychosocial factors experienced and explicitly mentioned by Mexican university students and teachers who performed their work and study activities from home in the 1st months of confinement due to the COVID-19 pandemic. The mixed technique of answers to open-ended inductors with the indication of the frequency of experiencing the expressed positive and negative aspects (Juárez et al., 2020) allowed for an inductive and grounded approach to analyze the semantic content, identifying emerging categories from the fieldwork (Glaser and Strauss, 1967; Corbin and Strauss, 2014) that could be transformed into quantitatively analyzable variables. The technique based on the proposal of Juárez and Camacho (2011) allowed us to identify aspects that fit into more conventional theoretical models and can be interpreted by them. For example, learning and personal development are appreciated (Bakker and Demerouti, 2014), while high quantitative and qualitative job demands are perceived as discomfort and social support counts (Karasek and Theorell, 1990; Demerouti and Bakker, 2023) but emerged as much more due to its positive presence or conflict potential in the domestic and family sphere than from organizational leaders or superiors. Nevertheless, showing the virtue of its inductive and explorative nature, the technique also allowed us to identify some aspects that reflect not only the contingency of the pandemic lockdown (such as lack of interaction) but also the advantages and disadvantages of working and studying from home in general (comfort, family interaction, noises, and distractors, etc.) and in the sociocultural context of countries like Mexico in particular (e.g., high relevance of family, infrastructural malfunction). Furthermore, this method allowed for basic statistical inferences and estimations despite not being as in-depth, purely qualitative research.

In this study, the emerging factors that reflected the answers of at least 10% of the sample were analyzed, standing out on the side of perceived positive aspects of studying and/or working from home, especially a positive *Family interaction* (25.93%), the possibility of *Self-management* (18.52%), and the benefit of *No need for commuting* (17.59%). These categories were followed by the answers of 15.74% of the sample into *Nutritional wellness*, the positive psychosocial factor with the highest average of being experienced (4.6 on a 1–5 scale). Particularly, positive relationships with the family resemble the findings of Al-Sabbah et al. (2021) for students and teachers in the Middle East during the COVID-19 lockdown, while negative family interactions or even conflicts were reported for university students by Robles et al. (2021) and Romeo et al. (2021) and for university teachers by Avila-Valdiviezo et al. (2021).

However, this research brought up the category of a negative *Family environment* in the answers of 10.19% of the participants. Nevertheless, regarding the negative aspects mentioned by most participants, the perceptions of *Work, school, and/or domestic activities overload* (26.85%) and problems in managing *Schedules* (23.15%) were particularly relevant, followed by the discomfort generated by *Online classes*, the inconvenience of *Interruptions, distractors, noise*, as well as *Problems with Internet services* (15.74% each). Finally, although only 10.19% of the participants mentioned *Stress* as a negative aspect of working/studying from home due to the COVID-19 confinement, it was the negative psychosocial factor with the highest mean frequency of being experienced (4.5 on a 1–5 scale), resembling the findings of Oleniuch (2021) and Charalampous et al. (2022) for people who worked from home during the pandemic, in general and of Robles et al. (2021) and Romero et al. (2022) for students, in particular.

The discomfort expressed by the participants about managing *Schedules* and *Online classes*, on the one hand, and their *Problems with Internet services*, on the other hand, could be seen as a reflection of the abrupt transition to homeschooling with neither training nor sufficient infrastructure, as pointed out by Alcántara (2020), Hurtado (2020), and Gazca-Herrera and Mejía-Gracia (2022), as well as of resulting technostress, especially for students (Martinez Arriaga et al., 2021; Robles et al., 2021; Gazca-Herrera and Mejía-Gracia, 2022; Romero et al., 2022; Villagrán et al., 2022). Nevertheless, it is worth mentioning that the answers of 14.81% of the participants in the present study mentioned *Adaptation and new learning* as positive challenges.

Either way, as just indicated above, the most prevalent emerging negative psychosocial factor was *Work, school*, and/or *domestic activities overload*. The qualitative work overload was also measured with the ESTC-COVID-19 scale, and the overall mean frequency of exposure was 3.46 (on a 1–5 scale) in the complete sample. The two variables correlated positively with a statistical significance at the 0.01 level, a result that strengthens the quality of the categorization of the open answers. Furthermore, the ESTC-COVID-19 scale correlated negatively with the positive psychosocial factor *Making better use of time*, indicating perhaps the importance of time management during the confinement period and the positive impact of achieving a containment of the inevitable work-life conflict when working from home (Lott and Abendroth, 2023).

Work intensification was observed in work at home even before the pandemic (Felstead and Henseke, 2017). According to other studies in Latin America, university teachers experimented with overload and incrementation of physical and mental demands during the pandemic (Robinet-Serrano and Pérez-Azahuanche, 2020; Godínez-Tovar et al., 2023). This study measured study and/or work workload and domestic tasks objectively by asking about the hours dedicated to it. Recalling a tendency for teachers to dedicate more hours per day to work and/or study and to domestic tasks than students could be observed in this study, it is important to contrast this result with the fact that it was the students who perceived more qualitative work overload in the ESTC-COVID-19 scale than the teachers.

Differences between students and teachers could also be observed in the perception of the emerging psychosocial factors. On average, teachers perceived the positive factor of *Self-management* more frequently and felt *Comfort* more often than students, and there is a statistically significant association between being a teacher and the emerging factor of *Enjoying home* during confinement. By contrast, being a student is associated with considering *Interruptions, distractors, noise* as a negative aspect of work and/or study from home, showing the problematic scholar invasion of the family sphere. For instance, the only psychosocial factor that represented more burden for teachers was *Domestic work*, since all the teachers who gave answers labeled with this category experimented with the frequency 5 = “always, every day.”

The fact that students seemed more negatively affected by studying and/or working from home is in line with the findings of Romeo et al. (2021), who observed that students suffer more from different negative aspects of COVID-19 confinement. The greater impact on students could be influenced by the different kinds of roles of being a student or a teacher. Specifically for students, the perception of the decrease in academic achievements could have a strong negative evaluation, either because they considered little attention from teachers or even because they saw their career path and future projects interrupted, as Romero et al. (2022) stressed out. In other words, learning, studying, and academic performance were affected. Furthermore, it should be further examined in subsequent studies whether self-management skills for one's own learning (as self-regulated learning essential for online education) were less developed in students (as perceived in this way causing reduced self-efficacy) as compared to independence, time, and task skills mentioned by teachers as a positive factor. Due to the nature of their respective roles, the latter might have been more developed at the time of the abrupt transition to virtuality.

In addition, since our sample had a very high female prevalence (76.85%), it did not seem convenient to analyze differences between women and men, but it seems important to remember that the spillover and blurring of boundaries between different life spheres during the confinement due to the COVID-19 pandemic, reflected in various emerging psychosocial factors discussed here, were particularly complicated to manage for women (Anwer, 2020; Sundari et al., 2020; Quezada et al., 2022b). In this sense, special attention from gender studies on work (and study) from home after the pandemic is also imperative.

Although COVID-19 restrictions no longer apply, some practices of distance schooling, teaching, and studying specifically and working from home remain and, in certain sectors, has even increased. Technological development and innovation opportunities allow and expand multiple possibilities of teleworking and virtual interaction. Although these can lead to better comfort, it is important to underline that the negative psychosocial aspects of working from home do not seem to diminish with time; on the contrary, they increase while benefits are reduced (Oleniuch, 2021) so that risk management, educational programs, and occupational health politics and practices should take into account the psychosocial factors that emerged during the pandemic. This is of utmost relevance, not only because of the exponential use of new virtual communicative forms in the teaching-learning process, but because, given the cost-saving results for organizations and companies, it establishes it as a new type of educational offer and a viable solution at eventual moments.

## Conclusion

In conclusion, the present study had several limitations. For instance, no differentiation between graduate and postgraduate students was possible, and the different work contracts of teachers (part-time vs. full-time, associates vs. tenures, etc.) could not be analyzed either. However, the study was cross-sectional, and its sample was not representative, so the results are hardly generalizable. Further, accountability of online surveys is less reliable than applications *in situ*. Moreover, the mixed technique allowed only basic statistical inferences, so it did not allow for a deepening nor an interactive, collaborative interpretation between researchers and participants. Nevertheless, the results showed grounded inductive emerging topics that mattered to people who responded to the survey, and its interpretation and discussion should be developed by future quantitative and qualitative research and informed public decision-making.

## Data availability statement

The raw data supporting the conclusions of this article will be made available by the authors, without undue reservation.

## Ethics statement

The studies involving humans were approved by Comité de Ética en Investigación del Centro de Investigación Transdisciplinar en Psicología CEI-CITPsi, CONBIOÉTICA-17-CEI-003-20190509. The studies were conducted in accordance with the local legislation and institutional requirements. The participants provided their written informed consent to participate in this study. Written informed consent was obtained from the individual(s) for the publication of any potentially identifiable images or data included in this article.

## Author contributions

BQ: Writing – review & editing, Writing – original draft. IH: Writing – review & editing, Writing – original draft. DC: Writing – review & editing, Writing – original draft.
